# Radiation Therapy in Management of Sporadic and Neurofibromatosis Type 1-Associated Malignant Peripheral Nerve Sheath Tumors

**DOI:** 10.3389/fonc.2014.00324

**Published:** 2014-11-17

**Authors:** Jenna Kahn, Andy Gillespie, Maria Tsokos, John Ondos, Eva Dombi, Kevin Camphausen, Brigitte C. Widemann, Aradhana Kaushal

**Affiliations:** ^1^Radiation Oncology Branch, National Cancer Institute, National Institutes of Health, Bethesda, MD, USA; ^2^Warren Alpert School of Medicine, Brown University, Providence, RI, USA; ^3^Pediatric Oncology Branch, National Cancer Institute, National Institutes of Health, Bethesda, MD, USA; ^4^Department of Pathology, National Institutes of Health, Bethesda, MD, USA

**Keywords:** malignant peripheral nerve sheath tumor, malignant schwannoma, neurofibrosarcoma, neurogenic sarcoma, radiation therapy

## Abstract

**Introduction:** Malignant peripheral nerve sheath tumors (MPNSTs) are highly aggressive soft tissue sarcomas in which complete surgical resection is the mainstay of therapy. However, the recurrence rate is high and few options remain for refractory or metastatic MPNST. This study examines the outcomes of adjuvant radiation therapy in MPNST in patients with and without neurofibromatosis type 1 (NF1) and reviews the literature on use of radiation for MPNST.

**Methods:** A retrospective review of 33 MPNST patients between 1990 and 2012 evaluated at the NIH. All diagnoses were pathologically confirmed at the NCI. Clinical presentation, treatment, and survival were analyzed.

**Results:** Thirty-three patients were included 18 NF1-associated, 15 sporadic tumors. Tumor location included extremity (58%), trunk (36%), and head/neck (6%). Histologic grade showed 25 high-grade tumors compared to 7 low-grade tumors. Twenty patients were treated with radiation therapy (median total dose of 58.5 Gy with 1.8 Gy/fraction). A median survival of all patients was 46.5 months and 43.7% overall 5-year survival. Prognostic factors include extent of resection, tumor location, and histology grade. Radiation was not found to be a prognostic factor for overall survival.

**Conclusion:** This study is consistent with previous studies regarding the role of radiation in the management of MPNST. Prospective evaluation of adjuvant radiation will allow to more fully define the role of radiation in MPNST.

## Introduction

Malignant peripheral nerve sheath tumors (MPNSTs), also known as malignant schwannomas, neurofibrosarcomas, and neurogenic sarcomas, are rare, highly aggressive malignancies that arise from major or minor peripheral nerve branches or sheaths. They account for about 10% of soft tissue sarcomas (1, 2). More than half of these malignancies arise in individuals with neurofibromatosis type 1 (NF1). The majority of NF1-associated MPNSTs arise from preexisting plexiform neurofibromas. The lifetime incidence of MPNSTs in NF1 patients is 8–13% whereas the general population has an incidence of 0.01% (1, 3–8). Several studies have shown that patients with NF1-associated tumors have a worse disease specific survival compared with sporadic tumors (1, 3, 9–12).

Complete surgical resection of MPNSTs is required for cure. Even with aggressive surgery and negative surgical margins, local and distant recurrence occurs frequently. Radiation and chemotherapy are additional modalities that are used to treat these tumors. However, poor response to standard sarcoma chemotherapy has been described for NF1-associated MPNST (1, 13). Despite current multimodality therapy, the 5-year survival ranges from 35 to 50% (1, 3, 6, 7). The role of radiation therapy in MPNST is still in evolution. Many studies have recommended the use of radiation therapy in an adjuvant setting (1, 3, 4, 10, 11, 14–19). Yet, only one study, to date, has shown a statistically significant increased local control with radiation therapy (10). Thus, the primary goal of this study was to examine the role and utility of adjuvant radiation therapy in patients evaluated at the NCI for sporadic and NF1-associated MPNST. A secondary goal was to review the literature of the use of radiation therapy in MPNST.

## Materials and Methods

Medical records of patients evaluated at the National Cancer Institute between January 1990 and June 2012 for potential diagnosis of MPNST were retrieved. For inclusion in this study, MPNST histological diagnosis was confirmed by a NCI pathologist. This was approved by the office of human subjects research protection as a retrospective analysis.

Malignant peripheral nerve sheath tumors were categorized as NF1-associated in patients with a clinical diagnosis of NF1 using consensus criteria or sporadic in the absence of a diagnosis of NF1 (20). Clinicopathologic data collected included age at initial diagnosis, gender, tumor location, tumor size, presence of metastases, extent of resection, histology grade, immunohistochemical staining for S100, and vimentin, neoadjuvant, and/or adjuvant chemotherapy, and/or radiation. Histology grade was defined as low or high grade. Low-grade referred to as grade I and high as grade II and III (21). Tumor location were defined as extremity (upper and lower), trunk (chest wall, proximal groin, thorax, abdomen, and retroperitoneum), or head and neck. Extent of resection were defined as R0 (negative margins), R1 (microscopically positive), and R2 (macroscopically positive) as defined in the pathology and surgical reports.

Radiation was defined as a treatment with curative intent. Radiation dose, fractions, field size, treatment breaks, and toxicities, resulting from radiation were also included. Acute toxicities were judged by radiation therapy oncology group (RTOG) criteria.

Overall survival (OS), the time from diagnosis to death, and the influence of clinicopathologic features on OS and local control was analyzed using the Kaplan–Meier method and using the log-rank (Mantel–Cox) test in the univariate setting. Statistical significance was defined as *p* < 0.05.

A comprehensive review of the literature for treatment of MPNST was conducted. The systematic literature search included Cochrane Collaboration Library electronic database, PubMed, RTOG.org, and ClinicalTrials.gov, using the following terms and keywords: MPNST, NF1, neurofibrosarcoma, malignant schwannoma, radiation therapy, and a combination of these terms. Studies were limited to those reported in the English language and human subjects. Original studies were reviewed independently.

## Results

### Patient and tumor characteristics

Of the 56 patients evaluated for MPNST, 33 fulfilled criteria for this study (Table 1). Most patients were excluded because there was not enough data or follow up. NF1-associated MPNSTs (*n* = 18) were more frequent than sporadic MPNSTs (*n* = 15). The median overall age at diagnosis was 25 years (range 9–76 years). There was a difference in median age between NF1 patients and sporadic, 15 and 41, respectively. Females constituted 33% (*n* = 11) of the study. There were more males in the NF1 study group as compared to the sporadic patients, 78 and 53%, respectively.

**Table 1:** **Patient characteristics**.

Characteristics	NF1 (*n* = 18)	Sporadic (*n* = 15)
Median age (years)	15	41
Gender
Female	4 (22%)	7 (47%)
Male	14 (78%)	8 (53%)
Presentation
Local	17 (94%)	14 (93%)
Metastatic	1 (6%)	1 (7%)
Tumor size (cm^3^)	931.4	275.6
Location of tumor
Head/neck	–	2 (13%)
Trunk	7 (39%)	5 (33%)
Extremity	11 (61%)	8 (53%)
Histology
Low grade	4 (22%)	3 (20%)
High grade	14 (83%)	11 (73%)
Presence of necrosis	9 (50%)	1 (7%)
S100
Positive	11 (61%)	10 (67%)
Negative	1 (6%)	2 (13%)
Vimentin
Positive	3 (17%)	8 (53%)
Negative	3 (17%)	2 (13%)
Extent of resection
R0	7 (39%)	6 (40%)
R1	4 (22%)	4 (27%)
R2	7 (39%)	5 (33%)
Neoadjuvant chemotherapy	2 (11%)	7 (47%)
Radiation	10 (56%)	10 (67%)
Treatment regimens
Surgery alone	4 (22%)	1 (6%)
Chemotherapy + surgery	4 (22%)	4 (27%)
Radiation + surgery	–	3 (20%)
Chemotherapy + surgery + radiation	10 (56%)	7 (47%)

Most tumors presented as local disease but one patient in each group presented with metastatic disease. NF1-associated tumors were larger at the time of diagnosis, 438.4 cm^3^ (range: 64–4377cm^3^), whereas the sporadic tumor volume was 240.6 cm^3^ (range: 10–1000 cm^3^). MPNSTs were most commonly located in the extremity (*n* = 19, 58%), followed by trunk (*n* = 12, 36%), and least commonly head and neck region (*n* = 2, 6%). Both NF1-associated and sporadic tumors were most common in the extremity, 61 and 53%, respectively.

Histologic grade of these tumors reported that high-grade disease was more prevalent (*n* = 25). The presence of necrosis was seen in nine patients with NF1 (50%) and one patients with sporadic tumors (7%). S100 positive staining was seen in 61% of NF1 patients while sporadic tumors showed 67% positive staining. Vimentin staining was positive in three patients with NF-associated and eight patients with sporadic tumors.

The extent of resection was similar in both groups. Negative surgical margins were achieved in 13 patients. R1 resection was achieved in 8 patients and R2 resection in 12. A total of 9 patients were treated with neoadjuvant chemotherapy and 20 patients (61%) treated with radiation therapy. The most commonly used chemotherapy agents for these patients included vincristine, adriamycin, cyclophosphamide, etoposide, docetaxel, ifosfamide, cisplatin, and gemcitabine. Treatment regimens consisted of surgery (*n* = 5), chemotherapy plus surgery (*n* = 8), radiation plus surgery (*n* = 3), and chemotherapy, radiation, and surgery (*n* = 17).

Twenty patients were treated with radiation therapy (Table 2). Modalities of radiation included external beam (*n* = 15), brachytherapy (*n* = 2), proton therapy (*n* = 1), and external beam plus brachytherapy (*n* = 2). NF1-associated tumors (*n* = 10) received a median total dose of 59.4 Gy with a median of 1.8 Gy/fractions. Sporadic tumors in the adjuvant setting (*n* = 10) received a median total dose of 58.5 Gy with a median of 1.8 Gy/fractions. Local control rates for NF-1 patients treated with radiation were 51% at 5 years. Local control rates for patients treated with radiation were 53% as compared to 45% in those not treated with radiation at 5 years, which was not statistically significant. Patients were treated with a mix of IMRT and 3D conformal using 6 MV. The field size of the tumor that was treated was substantially larger in NF1-associated tumors as compared to sporadic tumors, 597.9 cc^3^ (range: 31.5–2325.1 cc^3^) and 290.9 cc^3^ (range: 18–486.5 cc^3^), respectively. One patient from each NF1-associated and sporadic tumors required a treatment break during radiation. RTOG acute grade I toxicities were reported in a total of eight patients, three patients in the NF1 and five patients in the sporadic groups. Toxicities included fatigue, nausea, mild dysphagia, mild erythema, mild odynophagia, and desquamation. There were no toxicities exceeding grade II. Secondary cancers occurred in three patients including pilocytic astrocytoma, brain cancer (unspecified), and liver. Two of these patients had NF1 and the liver cancer was in the field of radiation treatment.

**Table 2:** **Radiation characteristics**.

Characteristics	NF1 (*n* = 10)	Sporadic (*n* = 10)
Type of radiation
External beam	7 (70%)	8 (80%)
Brachytherapy	1 (10%)	1 (10%)
Proton therapy	1 (10%)	0
External beam and brachytherapy	1 (10%)	1 (10%)
Adjuvant radiation
Fractions (median)	31	28
Dose/fraction (median)	1.8 Gy	1.8 Gy
Total dose (median)	59.4 Gy	58.5 Gy
Treatment volume (cc^3^) (mean)	438.4	240.6
Treatment breaks	1	1
RTOG acute grade toxicities	3	5

### Survival analysis

**Figure 1:**
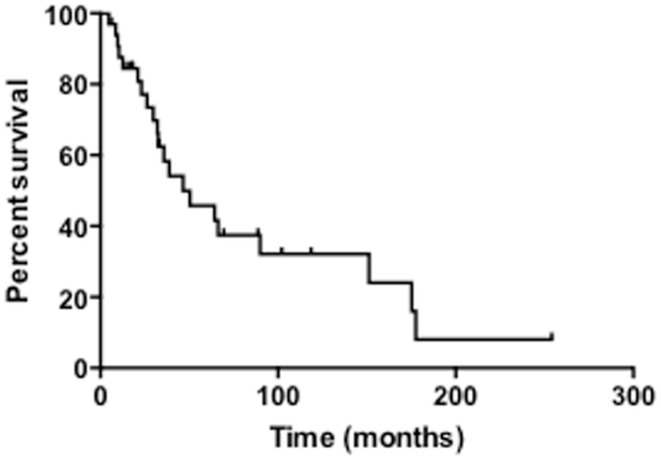
**Overall survival of patients with MPNST**.

**Figure 2:**
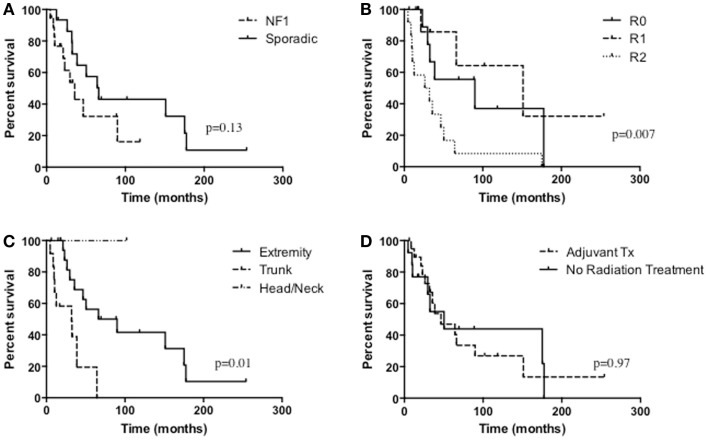
**Overall survival of (A) NF1-associated MPNST compared with sporadic MPNST, *p* = 0.13; (B) the extent of resection, *p* = 0.007; (C) tumor location, *p* = 0.01; (D) radiation treatment versus no radiation treatment, *p* = 0.97**.

The median survival of all patients was 33 months and overall 5-year survival was 43.7% (Table 3; Figure 1). In the NF1-associated tumors, the OS was 22.1 months as compared to sporadic tumors with a median of 64.3 months (*p* = 0.13) (Figure 2A). NF1-associated tumors treated with radiation had a median survival of 33.1 months as compared to 17.4 months with those patients that did not receive radiation treatment. Distant metastases accounted for five patients (28%) in NF1-associated MPNST as compared to seven patients in the sporadic MPNST (47%). Pulmonary metastasis was seen in three patients (17%) in the NF1-associated and seven patients (47%) in the sporadic MPNST. Poor prognostic factors that were statistically significant included incomplete resection (*p* = 0.007) (Figure 2B), histologic grade (*p* = 0.04), and truncal tumor location (*p* = 0.01) (Figure 2C). Radiation therapy was not found to be a prognostic factor for OS (*p* = 0.97) (Figure 2D).

**Table 3:** **Survival of NF1 and sporadic MPNST**.

Characteristics	NF1 (*n* = 18)	Sporadic (*n* = 15)
Overall survival (median)	22.1 months	64.3 months
Radiation	33.1 months	51.6 months
No radiation	17.4 months	69.4 months
Local failure	7 (39%)	5 (33%)
Distant failure	5 (28%)	7 (47%)
Pulmonary metastasis	3 (17%)	7 (47%)

### Literature review

The literature review identified 19 mostly retrospective studies published since 1973 with patients treated starting in 1920 until 2011. The studies included 20–205 patients with 17–100% with NF1. Tumor size as a prognostic factor for survival was significant in 12 studies where larger tumors had a worse prognosis. NF1 was regarded as a negative prognostic factor in seven studies. Age at initial diagnosis, where younger age was a worse prognosis for survival, was significant for four studies and tumor location for nine studies. Extent of resection was a prognostic factor in 14 studies, where incomplete resection had a worse survival prognosis. Radiation was a statistically significant local control prognostic factor in one study (10), yet, there was a trend and recommendation for radiation in seven reports. Overall of the 19 studies, 18–83% of patients received radiation therapy treatment. Doses ranged from 12.5–90 Gy in these studies. As seen in the table, OS ranged from 16 to 65.7% (Table 4).

**Table 4:** **Previous studies on treatment of MPNSTs**.

Reference	Institution	Dates	No. pts	NF1 (%)	Prognostic factors						Radiation		Overall survival (%)			
					NF1	Age	Tumor size	Tumor location	Extent of resection	Radiation	No. pts (%)	Dose (Gy)	5 year	10 year	5 year sporadic	5 year NF1
(9)	MSKCC	1920–1970	115	26	Y	–	–	–	Y	–	–	–	65.7	58.2	75	30
(14)	UCLA	1957–1977	20	70	N	–	–	–	Y	T	50	30–60	40	–	–	–
(22)	MSKCC		165	40	Y	N	N	Y	–	–	39	–	–	32	47	23
(3)	Mayo Clinic	1912–1983	120	52	Y	Y	Y	N	Y	N	49	–	34	22	53	16
(15)	MKSCC	1945–1988	43	53	N	–	Y	–	–	N	35	24–66.5	39	–	42	38
(4)	U Virginia HC	1960–1990	28	54	N	Y	Y	Y	Y	T	18	54–68	43.7	–	–	–
(2)	Netherlands	1977–1990	22	50	N	–	–	–	–	–	45	–	35	24	–	–
(23)	St. Jude’s	1964–1993	28	39	N	N	N	Y	Y	T	46	–	39	–	–	–
(10)	Mayo Clinic	1975–1993	134	24	Y	–	Y	Y	Y	Y	54	7.5–90	52	34	57	36
(18)	MKSCC	1960–1995	25	60	N	N	Y	N	Y	–	44	20–59.4	16	16	12	20
(17)	Italy	1976–1996	24	29	–	–	T	–	Y	T	50	50–54	–	–	–	–
(24)	London	1987–1995	29	100	N	N	Y	N	N	N	83	–	35	–	–	35
(16)	MGH	1991–2001	54	22	–	Y	Y	Y	Y	N	69	–	–	–	–	–
(1)	Italian and German	1975–1998	167	17	Y	Y	Y	Y	Y	T	63	45–70	51	43.4	55	32
(7)	Milan, Italy	1976–2003	205	22	T	N	Y	Y	Y	T	44	45–65	39.9	43.3	38.9	43.9
(25)	Germany	1991–2003	52	73	Y	N	N	Y	N	N	–	–	–	–	–	–
(6)	MDACC	1986–2006	140	55	N	–	Y	N	Y	–	49	50–56	38.7	26.4	42.3	34.8
(11)	Mayo Clinic	1985–2010	175	32	Y	–	Y	Y	Y	T	63	–	60	45	75	54
(26)	MKSCC	1982–2011	105	40	N	N	Y	N	Y	N	61	–	64 (3 years)	–	66 (3 years)	60 (3 years)
Present study	NCI	1990–2012	33	55	N	N	–	Y	Y	T	60.6	42–70.2	43.7	–	53.8	32.2

*T, trend toward yes but not statistically significant*.

*OS, overall survival*.

*Did not specify in paper*.

*Prognostic factors: NF1 (worse prognosis), age (younger worse prognosis), tumor location (extremities better prognosis), extent of resection (complete resection better prognosis), RT (Tx of radiation better prognosis)*.

## Discussion

Malignant peripheral nerve sheath tumors are uncommon soft tissue sarcomas. The prognosis for unresectable MPNST is poor. There is a scarcity of data addressing radiation therapy as a local therapy. Our study provides a detailed analysis of the treatment of patients with sporadic or NF1-associated MPNSTs as well as a review of the literature.

The association between MPNST and NF1 has been documented extensively. Studies have shown that individuals with NF1 have a higher than expected frequency of MPNST (3, 9, 10, 14, 27). This may be because patients with NF1 have a somatic mutation in the NF1 tumor suppressor gene, which results in the development of benign nerve sheath tumors called plexiform neurofibromas, which are at risk for malignant degeneration (28). In many studies, the mean age of diagnosis of MPNST in these NF1 patients is between 25 and 28 years old (2–4). As seen in the literature age at diagnosis has also been a prognostic factor for some studies including Wanebo et al. who found that there was reduced survival in patients younger than 30 years of age reflecting on the aggressiveness in the younger population (4). Our study similarly showed a median diagnosis of 15 years old. In sporadic tumors, the mean age of diagnosis for patients has ranged from 39 to 60 in studies (2–4). Our study likewise shows a median of 41 years old for sporadic tumors.

Some studies have shown that NF1 was a poor prognostic factor for survival statistically while others have shown a trend toward sporadic tumors being a good prognostic indicator (1, 3, 9–12, 15, 18, 22). The 5-year survival rates of those with NF1-associated tumors ranged from 16 to 60%, whereas in sporadic tumors the rates ranged from 47 to 75% (1, 3, 9–11, 16, 22). At diagnosis, NF1-associated MPNST tend to be large, invasive, and unresectable (1). NF1-associated MPNSTs are commonly more frequent in the trunk as compared to sporadic MPNST (6, 11), which affects outcome because central lesions may be less amenable to surgery than are extremity MPNSTs (3, 22). NF1-associated MPNSTs have also been thought to have a greater tendency to metastasize than sporadic MPNSTs. Ducatman et al. showed that of 62 patients with NF1-associated MPNSTs, 39% developed metastatic disease whereas only 16% of 58 sporadic MPNSTs developed metastases (3). The diagnosis of MPNST in patients with NF1 is difficult to establish clinically without biopsy because they are often mistaken for neurofibromas and therefore present at a later stage (3, 15, 29). NF1-associated tumors had a 5-year OS of 32.2% as compared with 53.8% in sporadic tumors. Although our data does not show statistical significance (*p* = 0.13) for poor prognosis of NF1 patients our data does show a trend that sporadic patients have a better prognosis. Our data correlate with these studies and suggest that the need for expeditious evaluation in patients with NF1 with rapidly growing lesions.

Tumor location has been hypothesized as a prognostic factor because of the ability of complete surgical resection to be more easily achieved in extremities versus tumors in the abdomen or chest (3, 22). Primary tumors of the extremities are also thought to be diagnosed earlier because the more visible location (10, 22). Tumor location was found to be a prognostic factor in nine studies with extremities having the better prognosis. The location of the tumor was found to be a prognostic factor for survival in our study (*p* = 0.01). Tumors located in the truncal region fared worse than those with lesions in the extremities, which is consistent with the literature.

Malignant peripheral nerve sheath tumors are locally aggressive and failure to achieve local control remains the major cause of treatment failure. Complete surgical resection is the only curative treatment for sporadic and NF1-associated MPNST (1). The surgical goal is to resect the tumor with wide negative margins. In our study as well as previous studies, incomplete resection and negative margins have been shown to be statistically significant for survival (1, 3, 4, 6, 7, 9–11, 14, 16–18, 23, 26). The results of this study indicate that complete surgical removal of the tumor is the mainstay of treatment and a strong predictor of survival.

Multidisciplinary evaluation of these patients is crucial in order to provide optimal care. Numerous studies have not found radiation therapy to be a prognostic factor for local control or survival (1, 3, 4, 6–8, 11, 12, 15, 16, 18, 22–25). Yet, many studies have also stated that the use of adjuvant therapy is expanding when patients do not have clear surgical margins (1, 4, 7, 30). Although there have been no prospective randomized trials of radiation therapy in the context of MPNST, there have been studies of the role of adjuvant radiation in adult soft tissue sarcomas (31, 32). Yang et al. found that post-operative radiation therapy is highly effective in local control in soft tissue sarcomas of the extremities (31). Wong et al. found that the 5-year local control rates for radiation doses higher than 60 Gy was 73% as compared with 50% for lower doses (*p* < 0.021), showing that post-operative radiation therapy has a role in improving local control (10, 17). Yet, in an updated study of Stucky et al. with some of the patients from the Wong et al. study as well as two more institutions, the analysis showed radiation therapy not to be a prognostic factor for local control. It was hypothesized that this may be because of an evolution of treatment with an emphasis of achieving negative margins. Even so, Stucky et al. encouraged that radiation therapy be utilized for tumors that have aggressive features including size ≥5 cm, high grade, and R1 or R2 margin status (11). Our study similarly showed that the patients treated with adjuvant radiation were not a prognostic factor for local control. This study should also take into account that there is an inherent selection bias given the retrospective nature of the study. Patients were likely selected for radiation therapy based on more extensive tumors, margin status, and unresponsiveness to other therapies, which would bias the results toward not showing a benefit of radiotherapy. As evident by these studies, the role of radiation therapy is still being defined.

In this series, we evaluated the effectiveness of radiation therapy on survival in the setting of MPNST. Radiation therapy treatment was not statistically significant for OS in our study. These data are consistent with the literature where there is no MPNST study that shows a statistically significant difference in OS for those with radiation treatment. In a SEER analysis, high-grade tumors treated with radiation did have improved survival where they looked at 142 patients with MPNST and grouped them with other soft tissue sarcomas (33). Although there have not been studies only looking at MPNSTs, this study would infer that high-grade soft tissue sarcomas, including MPNST, should be treated with radiation therapy. Aggressive therapeutic approaches are important in patients at high risk and every effort should be considered.

In our study, most patients treated with radiation received external beam therapy while few patients received protons and brachytherapy. In the adjuvant setting, the total radiation dose was ≥56 Gy. This is compared with previous studies, which have found that a cumulative dose of ≥60 Gy was required to provide local disease control (10, 32). Although very few patients were treated with brachytherapy this modality was given as a boost to external beam radiation. Previous studies have evaluates the use of brachytherapy for soft tissue sarcomas and found it to be an effective treatment in combination with external beam radiation in local control (34). One study, in particular, found the use of brachytherapy or intraoperative electron radiation therapy to be a prognostic factor for local control with a 5-year local control of 88% in those treated with brachytherapy and 51% in those treated with external beam (10). Adjuvant external beam therapy in another study was found to have better local control then brachytherapy (35). Only one patient in our study received proton therapy and is alive without evidence of disease. Even though there have been no studies on proton beam therapy in MPNSTs this modality of treatment may be a viable option for younger patients or to minimize toxicity.

## Conclusion

In conclusion, our analysis shows that radiation therapy can be effective in achieving local and symptomatic control with well-tolerated toxicities. Prognostic factors for survival include tumor location, histology, and extent of surgical resection. Our study is limited by its retrospective nature, small sample size, and heterogeneity in the patient population analyzed and their treatment. Prospective studies evaluating the effect of radiation therapy is required. In the adjuvant setting, it will be imperative to assess the dose and modality of treatment. The role of radiosensitizers and other novel techniques should also be explored.

## Conflict of Interest Statement

The authors declare that the research was conducted in the absence of any commercial or financial relationships that could be construed as a potential conflict of interest.
